# Novel Variant in *CEP250* Causes Protein Mislocalization and Leads to Nonsyndromic Autosomal Recessive Type of Progressive Hearing Loss

**DOI:** 10.3390/cells12182328

**Published:** 2023-09-21

**Authors:** Minjin Kang, Jung Ah Kim, Mee Hyun Song, Sun Young Joo, Se Jin Kim, Seung Hyun Jang, Ho Lee, Je Kyung Seong, Jae Young Choi, Heon Yung Gee, Jinsei Jung

**Affiliations:** 1 Department of Otorhinolaryngology, Graduate School of Medical Science, Brain Korea 21 Project, Yonsei University College of Medicine, Seoul 03722, Republic of Korea; mjkang@yuhs.ac (M.K.); jychoi@yuhs.ac (J.Y.C.); 2 Institute for Lee Won Sang Yonsei Ear Science, Seoul 03722, Republic of Koreakin2844@yuhs.ac (S.Y.J.);; 3 Department of Pharmacology, Graduate School of Medical Science, Brain Korea 21 Project, Yonsei University College of Medicine, Seoul 03722, Republic of Korea; 4 Department of Otorhinolaryngology Head and Neck Surgery, Myongji Hospital, Hanyang University College of Medicine, Goyang 04763, Republic of Korea; 5 Graduate School of Cancer Science and Policy, National Cancer Center, Goyang-si 10408, Republic of Korea; ho25lee@ncc.re.kr; 6 Laboratory of Developmental Biology and Genomics, BK21 PLUS Program for Creative Veterinary Science Research, Research Institute for Veterinary Science, College of Veterinary Medicine, Seoul National University, Seoul 08826, Republic of Korea; snumouse@snu.ac.kr

**Keywords:** genetic hearing loss, *CEP250*, *C-Nap1*, centrosome, ciliary protein

## Abstract

Genetic hearing loss is the most common hereditary sensorial disorder. Though more than 120 genes associated with deafness have been identified, unveiled causative genes and variants of diverse types of hearing loss remain. Herein, we identified a novel nonsense homozygous variant in *CEP250* (c.3511C>T; p.Gln1171Ter) among the family members with progressive moderate sensorineural hearing loss in nonsyndromic autosomal recessive type but without retinal degeneration. *CEP250* encodes C-Nap1 protein belonging to the CEP protein family, comprising 30 proteins that play roles in centrosome aggregation and cell cycle progression. The nonsense variant in *CEP250* led to the early truncating protein of C-Nap1, which hindered centrosome localization; heterologous expression of *CEP250* (c.3511C>T) in NIH3T3 cells within cilia expression condition revealed that the truncating C-Nap1 (p.Gln1171Ter) was not localized at the centrosome but was dispersed in the cytosol. In the murine adult cochlea, Cep250 was expressed in the inner and outer hair cells. Knockout mice of *Cep250* showed significant hair cell degeneration and progressive hearing loss in auditory brainstem response. In conclusion, a nonsense variant in *CEP250* results in a deficit of centrosome localization and hair cell degeneration in the cochlea, which is associated with the progression of hearing loss in humans and mice.

## 1. Introduction

Hereditary hearing loss is the most common sensorial disease worldwide [1]. In half of the congenital hearing loss cases, the cause is genetic defects, two-thirds of which are classified as nonsyndromic hearing loss (NSHL) [2]. To date, more than 120 genes associated with NSHL have been found and more than 400 syndromic disorders have been identified (http://hereditaryhearingloss.org/, accessed on 1 June 2023). Over the past decade, whole-exome sequencing (WES) has been used to identify pathogenic genetic variants that cause hearing loss [3,4] and to discover many novel deafness genes [3,5,6]. However, there are still cases of hearing loss inheritance in which the genetic causes have not been identified, indicating that there remain unveiled genetic causes of hearing loss that need to be clarified.

*CEP250* encodes the C-Nap1 protein that belongs to the CEP protein family, which includes more than 30 proteins that form active centrosomal components and play important roles in centrosome aggregation and cell cycle progression [7,8]. C-Nap1 is expressed in photoreceptor cilia and interacts with other ciliary proteins, including Rootletin and NEK2 [9]. A study on retinal cilia formation in ARPE19 cells reported that *CEP250* is involved in cilia formation [10]. It is known that nonsense mutations in *CEP250* cause atypical Usher syndrome, characterized by early onset sensorineural hearing loss and relatively mild retinitis pigmentosa (RP) [11,12]. Furthermore, some nonsense variants in *CEP250* are associated with only mild cone dystrophy and late-onset sensorineural hearing loss [13,14]. One study reported that missense variants in *CEP250* are associated with nonsyndromic RP, indicating that the phenotype varies depending on the type and severity of variations in *CEP250* [10].

Herein, we report, for the first time, that a novel nonsense variant of *CEP250* was identified in a family with progressive hearing loss but with no ocular symptoms, representative of NSHL. In the present study, we found that the nonsense variant of *CEP250* caused the mislocalization of truncated C-Nap1 from the centrosome. In addition, we observed hair cell degeneration and adult-onset progressive hearing loss in *Cep250* knockout mice, which is consistent with human data.

## 2. Materials and Methods

### 2.1. Patients and Diagnosis of Genetic Hearing Loss

Patients with hearing loss of unknown origin were enrolled in the Yonsei University Hearing Loss (YUHL) cohort. All patients registered in the YUHL cohort had hearing loss and were referred to Severance Hospital for further evaluation and treatment, as previously reported [15]. Briefly, pure-tone and speech audiograms were obtained for all patients and their affected and unaffected family members. Pure-tone air (250–8000 Hz) and bone conduction (250–4000 Hz) thresholds were measured using a clinical audiometer in a double-walled audio booth. The degree of hearing loss was determined by averaging the air-conduction thresholds at 500, 1000, 2000, and 4000 Hz. The auditory steady-state response was also determined in young babies. Temporal bone computed tomography and magnetic resonance imaging were performed to evaluate inner ear abnormalities.

### 2.2. WES and Analyses

WES was performed as previously described [16,17]. DNA was extracted from each member’s blood sample, WES was performed using a sequencing system (Hiseq 2500), and the variants were identified by comparison with the human reference genome (GRCh37/hg19) and filtered. Variant calling detected 32,912 variants. After removing variants with a count of less than 3 or a frequency of less than 25, 19,567 variants remained. After excluding variants that were previously reported in the Deafness Variation Database (https://deafnessvariationdatabase.org/, accessed on 10 July 2023) or ClinVar (https://www.ncbi.nlm.nih.gov/clinvar/, accessed on 10 July 2023) or had been determined as benign in the YUHL cohort, 5224 variants remained. Of the total, 641 variants were identified as non-synonymous or obligatory splice site variants. According to the expert-specified American College of Medical Genetics and Genomics/Association for Molecular Pathology (ACMG/AMP) guideline [18], we chose rare variants with allele frequencies below 0.005 based on total or East Asian data in the genome aggregation database (gnomAD), resulting in 388 variants. Given the autosomal-recessive pattern of inheritance observed in the proband, we focused on 33 variants that were suspected to be homozygous or compound-heterozygous. We examined these variants using the Integrative Genomics Viewer and excluded variants with potential false alignments or poor sequence quality. Additionally, two variants located in cis, which were inherited from one of the parents, were also excluded. Subsequently, nine variants in six genes remained (Supplementary Table S1). Finally, the remaining variants were ranked based on the following criteria: whether mutations were truncating the conceptual reading frame (non-sense, frameshift, and obligatory splice variants), the results of an evolutionary conservation analysis of missense variants, and predictions of the potential disease impact of candidate variants on the encoded protein using web-based programs. Additionally, known disease-causing mutations were considered in the ranking process. Online Mendelian Inheritance in Man (OMIM) database analysis revealed that among the six remaining genes, *CEP250* was the only gene associated with cone-rod dystrophy and hearing loss, thus establishing its potential causative role.

### 2.3. pRK5-Myc-CEP250 Recombinant Vector Construction and Mutagenesis

*CEP250* cDNA was reverse-transcribed from human mRNA. Complete *CEP250* cDNA (HsCD00399261) was subcloned into pRK5-Myc to construct the recombinant vector pRK-Myc-CEP250. The vectors were tagged with Myc and Ampicillin. The *CEP250* clone (prk5Myc-CEP250, insert:7329 bp, vector:4754 bp) was used to construct a clone with the same mutation as that of *CEP250* (p.Gln1171Ter) found in YUHL251-21. In the wild type [5′-CGC AGA GCA GCA GCC CGG GAA-3′] nucleotide sequence, to replace nucleotide C at position 3511 with T, mutant type [sense, 5′-CGCAGAGCAGTAGCCCGGGAA-3′] and [antisense, 5′-TTCCCGGGCTACTGCTCTGCG-3′] primers were prepared. After obtaining the substituted product through a polymerase chain reaction, plasmid DNA extraction (QIAgen, QIAprep spin miniprep kit) was performed to obtain a purified plasmid. The presence of these mutations was verified by Sanger sequencing.

### 2.4. Cell Culture and Transfection

NIH3T3 cells were maintained in DMEM (Dulbecco’s Modified Eagle’s Medium, 4.5 g/L glucose, and L-glutamine, Lonza, 12-604F) supplemented with 10% FBS (fetal bovine serum; Gibco, 16000044) and penicillin (50 IU/mL)/streptomycin (50 μg/mL) at 37 °C with 5% CO_2_. The expression plasmids for pRK5-Myc-CEP250-WT or pRK5-Myc-CEP250-p.Gln1171Ter (c.3511 C>T) transfection of each plasmid into NIH3T3 cells was performed using JetPRIME reagent (Polyplus, 114-15).

### 2.5. Immunoblotting

Immunoblotting was performed as previously described [19]. The adherent cells were scraped from the dish using a cold plastic cell scraper, and the cell suspension was transferred into an Eppendorf tube. Cell samples were sonicated in a lysis buffer. Whole-cell lysates were centrifuged in a microcentrifuge at 13,000× *g* for 20 min at 4 °C. The tube was then removed from the centrifuge, and placed on ice. Subsequently, the supernatant was aspirated, placed in a new tube kept on ice, and the pellet was discarded. Lysed samples were mixed with sample buffer and separated by sodium dodecyl sulfate (SDS)-polyacrylamide gel electrophoresis. The separated proteins were transferred to a nitrocellulose membrane and blotted with the appropriate primary and secondary antibodies. Protein bands were detected using enhanced chemiluminescence (GE Healthcare, RPN2109, Hatfield, UK).

### 2.6. Immunocytochemistry

The NIH3T3 cells were cultured on coverslips and were maintained in DMEM (Dulbecco’s Modified Eagle’s Medium, 4.5 g/L glucose, and L-glutamine, Lonza, 12-604F, Basel, Switzerland) supplemented with penicillin (50 IU/mL)/streptomycin (50 ug/mL) at 37 °C with 5% CO_2_ and incubated for 30 h. After washing with phosphate-buffered saline (PBS; 17-516F; Lonza), cells were treated with 4% paraformaldehyde (PFA; PC2031-100-00; Biosesang, Yongin-si, Republic of Korea) for 10 min at room temperature (RT). Then, they were treated with 0.1% SDS (50 mL PBS, 0.05 g SDS, Sigma, 436143-25G) at RT for 10 min. To block nonspecific binding sites, the cells were incubated with blocking media (0.2 mL PBT containing 5% normal goat serum and 1% BSA) at RT for 1 h. The cells were stained by incubation with the appropriate primary and secondary antibodies and viewed under an LSM700 confocal microscope (Zeiss Laboratories, Munich, Germany).

### 2.7. Cep250 Knockout Mouse

C57BL/6J mice and *Cep250* knockout mice were used in our experiments. The *Cep250* knockout mice (C57BL/6N-Cep250^em1cyagen^) were purchased from Cyagen (Santa Clara, CA, USA), and *Cep250* knockout mice were generated by CRISPR/Cas9 strategy. The guide RNA sequences used to target *Cep250* were as follows: CAGAAACTCTTAAGTCGCTGTGG, CATTACCTAGTGGAATTTGCAGG, GCAAGTT-TGTCCAAGATTCTTGG, and AAGAGACTTACCTGTTCTGGTGG. Deletion of 8849 bp, from exon 3 to exon 12, was detected in the knockout allele.

### 2.8. Inner Ear Immunoblotting

Inner ears were dissected from approximately 7-week-old C57BL/6J mice and *Cep250* knockout mice. Ear tissue samples were chopped and lysed in lysis buffer. After centrifugation, the supernatant was identical to that used for the immunoblotting procedure described above.

### 2.9. Inner Ear Immunohistochemistry

Mouse inner ear immunohistochemistry was performed as previously described [20]. Briefly, mouse inner ear tissue samples were fixed in 4% PFA at 4 °C and then washed twice with PBS. Specimens were calcified in 25% ethylenediaminetetraacetic acid/PBS for 24 h. Tissue samples were dehydrated and embedded in paraffin for histological studies. Samples were then cut into segments with the organ of Corti and permeabilized with 0.1% Triton X-100 for whole-mount immunostaining. Paraffin blocks were sectioned into 5 μm using a microtome (Leica Biosystems, Shinjuku City, Tokyo). Subsequently, the tissue sections were immunostained. The paraffin sections were deparaffinized with xylene, ethanol, and PBS; for antigen retrieval, they were incubated in sodium citrate at 95 °C for 5 min and then blocked in 10% normal donkey serum for 1 h at RT. Tissue samples were incubated overnight with target-specific primary antibodies at 4 °C. The samples were then washed and incubated with the appropriate secondary antibodies for 1 h at RT. The samples were washed and mounted with mounting solution (Sigma, St. Louis, MO, USA). All immunostained images were obtained using a confocal microscope LSM700 (Zeiss Laboratories, Chiyoda City, Tokyo). The whole-mount preparation was performed as previously reported.

### 2.10. Auditory Brainstem Response Test

Auditory brainstem response (ABR) tests were performed as previously reported [21]. Briefly, the ABR thresholds were measured in a soundproof chamber using Tucker-Davis Technologies (TDT) RZ6 digital signal processing hardware and BioSigRZ software (Alachua, FL, USA; https://www.tdt.com/component/biosigrz-abr-dpoae-software/). Subdermal needles (electrodes) were positioned at the vertex, ventrolateral to the right and left ears of anesthetized mice. A calibrated click stimulus (10 µs) or tone burst stimulus (5 ms) was produced at 6, 12, 18, 24, and 30 kHz using SigGenRZ software (https://www.tdt.com/docs/siggenrz/introduction/#what-is-siggenrz) and an RZ6 digital signal processor. These stimuli were then delivered to the ear canal using a multi-field 1 (MF1) magnetic speaker (TDT). The recorded signals were filtered using a 0.5–1 kHz band-pass filter, and the ABR waveforms in response to 512 tone bursts were averaged. The ABR thresholds for each frequency were determined using BioSigRZ software.

### 2.11. Statistical Analysis

The results of multiple experiments are presented as the means ± SEM. Statistical comparisons were performed using Student’s *t*-test. The data were compiled using Microsoft Excel. *p* < 0.05 was considered statistically significant.

## 3. Results

### 3.1. Identification of CEP250 as a Candidate Deafness Gene for High-Frequency Sensorineural Hearing Loss

**Figure 1:**
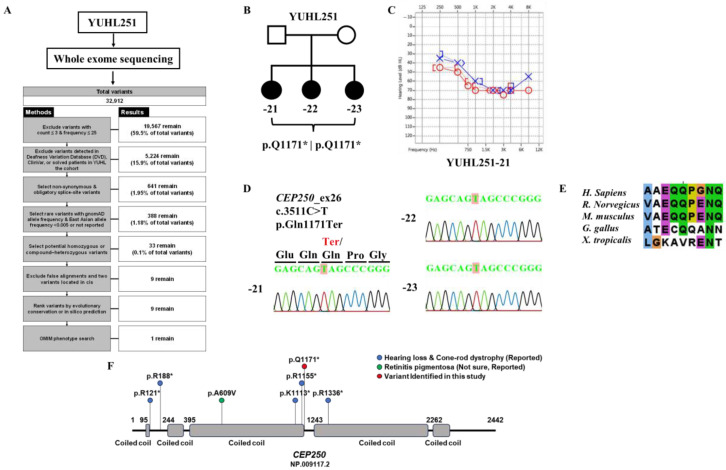
Discovery of new causative gene CEP250 mutations in patients with hearing loss through whole-exome sequencing methods. (**A**) Whole-exome sequencing analysis in the YUHL cohort was performed and *CEP250* was found as a novel deafness gene in YUHL 251 family. (**B**) Pedigree and found mutations of YUHL251 (−21, 42-year-old female; −22, 40-year-old female; −23, 35-year-old female. (**C**) Hearing tests showed high-frequency sensorineural hearing loss in YUHL251-21. Red line (circle; air conduction, [ or <; bone conduction] refers to the right ear threshold and blue line (cross; air threshold,] or >; bone conduction) refers to the left ear threshold. (**D**) The autosomal recessive *CEP250* c.3511C>T, p.Gln1171Ter gene mutation was discovered through whole-exome sequencing. (**E**) Evolutionary conservation of altered amino acid residues (*Homo sapiens* (human), *Rattus norvegicus* (rat), *Mus musculus* (mouse), *Gallus gallus* (chicken), *Xenopus tropicalis* (frog)). (**F**) Schematic diagram of pathogenic variants in *CEP250*. Abbreviations: YUHL, Yonsei University Hearing Loss. Asterisk (*) denotes a stop codon.

In the YUHL cohort dataset comprising patients with hereditary hearing loss, sensorineural hearing loss was observed in YUHL251-21 (Figure 1C). The proband (YUHL251-21) showed moderate sensorineural hearing loss with an onset age of late 30 s. The inheritance pattern was autosomal recessive. All affected individuals had normal visual acuity without night blindness. In addition, they did not have any problems with the spectral-domain optical coherence tomographic images, which was confirmed by ophthalmologic examination. In the whole-exome sequencing data, a homozygous nonsense variant of CEP250 (c.3511C>T) was identified in YUHL251-21 (Figure 1A,B). In the segregation analysis, Sanger sequencing revealed that the affected siblings of YUHL252-22 and -23 had a homozygous nonsense variant of *CEP250*. It was confirmed that the mutation in exon 26 of the gene changed to C>T at c.3511, resulting in the termination of the amino acid glutamine (Figure 1D). The position of p.Q1171 was well conserved across species (Figure 1E). The reported pathogenic variants in *CEP250* are shown in Figure 1F.

### 3.2. Expression of CEP250 WT and p.Gln1171Ter Variant in NIH3T3 Cells

**Figure 2:**
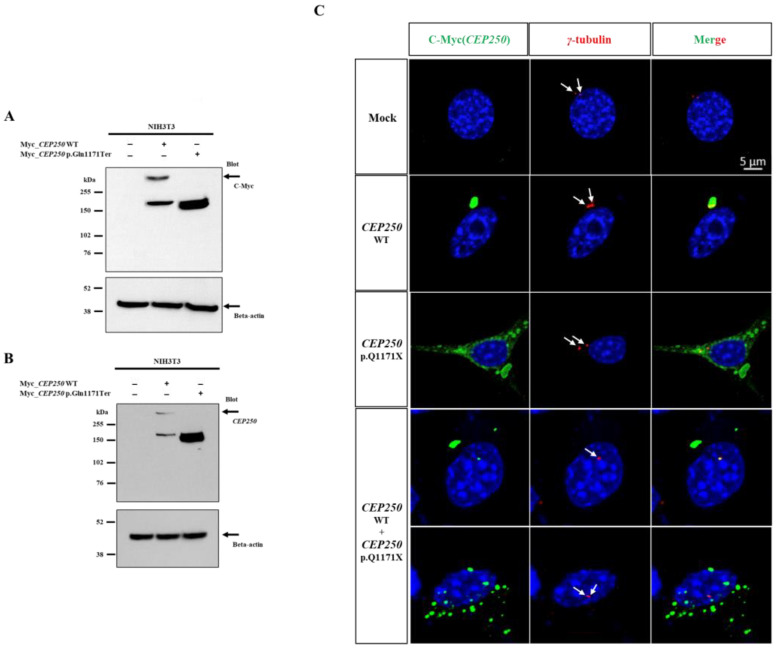
Expression of p.Gln1171Ter C-Nap1 in NIH3T3 cell line. (**A**,**B**) Immunoblotting analysis using Myc (**A**) and CEP250 (**B**) antibodies. *CEP250* WT was expressed at 255 kDa (full length) and ~160 kDa (short form). CEP250 p.Gln1171Ter was detected at ~150 kDa, corresponding to the early truncated protein. (**C**) Immunocytochemistry was performed in NIH3T3 cells transfected with wild-type CEP250 and p.Gln1171Ter. It was stained using γ-tubulin (red), Dapi (blue), and C-Myc (green) antibodies in NIH3T3 cells. White arrows point to centrosome.

We investigated the expression of the early truncated CEP250 protein in the NIH3T3 cell line. *CEP250* wild-type and p.Gln1171Ter variants were transfected into NIH3T3 cells under conditions of ciliary formation to determine whether the truncated protein had any deficit in cellular expression. In Western blotting, the Myc antibody detected an expected band at 250 kDa and an additional band at 150 kDa in WT *CEP250* (Figure 2A,B). In contrast, the antibody detected only one band at 150 kDa for the *CEP250* p.Gln1171Ter variant.

Next, we performed immunocytochemistry to investigate the localization of mutant CEP250. The samples were immunostained with anti-γ-tubulin to detect centrosomes. CEP250 WT co-localized well at the centrosome, whereas CEP250 p.Gln1171Ter did not localize at the centrosome and was dispersed throughout the cell cytosol (Figure 2C). When we performed the co-expression of the wild type and p.Q1171X Cep250, both co-localized at the centrome and dispersed Cep250 in the cytosol were observed, indicating that p.Q1171X Cep250 does not have a dominant negative effect on wild-type Cep250. Taken together, the *CEP250* c.3511C>T nonsense variant was not subject to nonsense-mediated decay and could be translated into the early truncated protein. However, it failed to localize at the centrome, indicating that the nonsense variant may lose the capability of centrosome cohesion.

### 3.3. Effect of CEP250 p.Gln1171Ter Variant on the Cilia Development in NIH3T3 Cells

**Figure 3:**
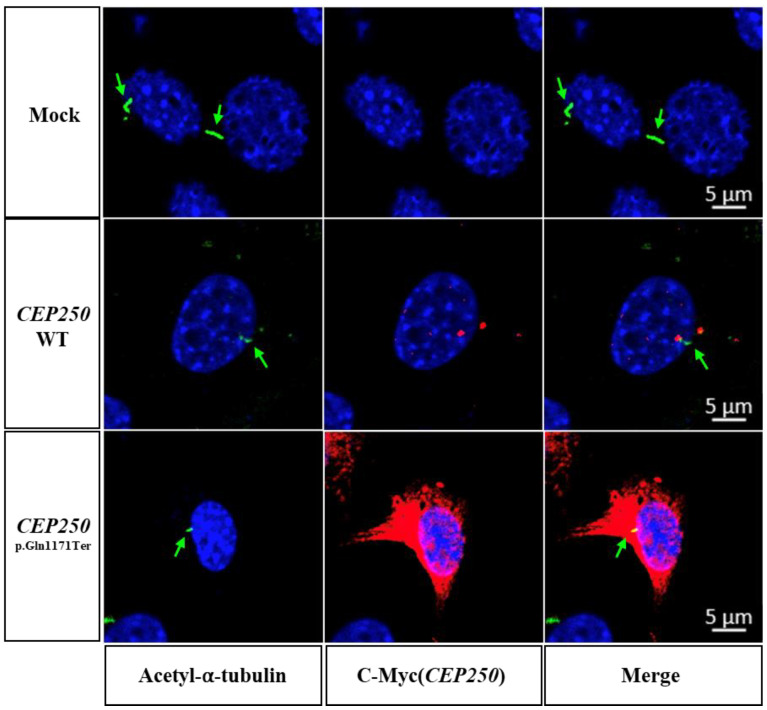
Cilia expression in NIH3T3 cells transfected with CEP250 and p.Gln1171Ter variant in immunostaining. Staining with Acetyl-⍺-tubulin (green), C-Myc (red, CEP250) antibody to confirm whether the expression of CEP250 p.Gln1171Ter mutation affects the production and growth of primary cilia. Both CEP250 WT and p.Gln1171Ter mutations confirmed the production of primary cilia. Green arrows point to primary cilia.

We investigated whether ciliary development was affected by the CEP250 p.Gln1171Ter variant to rule out the possibility of a dominant-negative effect. As the NIH3T3 cell does not have endogenous CEP250 (Figure 2B), it was possible to observe the independent overexpression effect of the CEP250 WT or p.Gln1171Ter variant on ciliary development. When the medium was serum-free for 30 h in the NIH3T3 cell line, the cell cycle was arrested from G1 and transitioned to G0 to induce cilia [22]. When CEP250 WT or p.Gln1171Ter was overexpressed in NIH3T3 cells, there was no effect on ciliary length compared with the control (Figure 3). This indicates that CEP250 is not necessarily required to elongate primary cilia and that early truncation of CEP250 does not have any dominant negative effect on ciliary development.

### 3.4. Cep250 Is Expressed in the Cochlear Hair Cells and Spiral Ganglion

**Figure 4:**
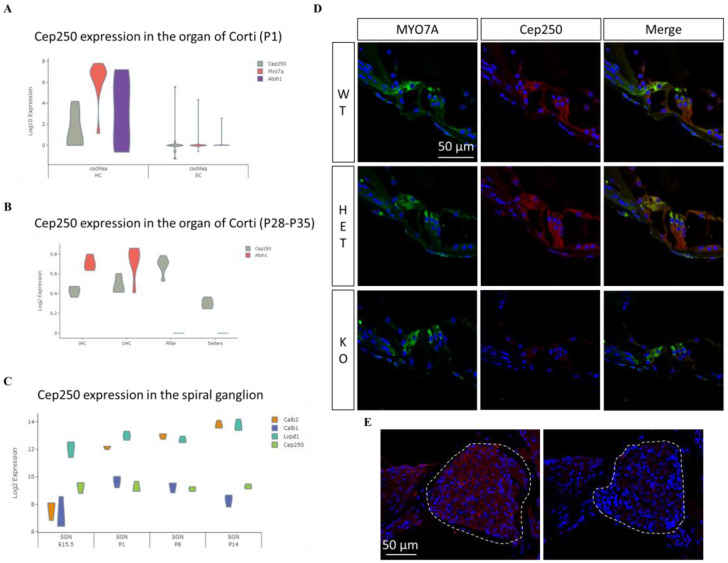
Expression and localization of Cep250 in the mouse cochlea. (**A**,**B**) *Cep250* transcriptional expression in the organ of Corti at P1 (**A**) and P30 (**B**). *Myo7a* and *Atoh1* are depicted as hair cell markers. HC, hair cell; SC, supporting cell. (**C**) *Cep250* transcript expression in the spiral ganglion neuron at different ages. *Calb2*, *Calb1*, and *Lypd1* are markers for type I, II, and III neurons, respectively. SGN, spiral ganglion neuron. (**D**,**E**) Immunohistochemistry was performed for wildtype and *Cep250* knockout mice (P30). Cep250 (red) was expressed in the inner hair cells (IHCs), outer hair cells, and pillar cells in the organ of Corti of wildtype mice but not *Cep250* knockout mice (**D**). Cep250 (red) is expressed in the spiral ganglion (dashed area) of wildtype mice but not in that of the *Cep250* knockout mice (**E**). Dapi (blue) staining. WT, wildtype; Het, heterozygous *Cep250* knockout mice; KO, homozygous *Cep250* knockout mice.

To identify the expression and localization of Cep250 in the cochlea, we first evaluated the transcriptional level of *Cep250* in the cochlea from the public gene expression analysis resource (gEAR, https://umgear.org/, accessed on 31 Aug 2023) [23]. The transcriptional level of *Cep250* was higher in the hair cells than in supporting cells at P1 (Figure 4A) [24]. However, Cep250 expression was also found in the pillar cells and supporting cells (Deiters’ cells) at adult stages (Figure 4B) [25]. In spiral ganglion neurons, Cep250 is consistently expressed from E15.5 to P14 (Figure 4C) [26]. Next, we performed immunohistochemistry in the cochlea of wildtype and *Cep250* knockout adult mice. Cep250 was identified in the inner hair cells, outer hair cells, pillar cells, supporting cells, and spiral ganglion neurons in the wildtype mice but not in the *Cep250* knockout mice (Figure 4D,E).

### 3.5. Hearing Loss in the Cep250 Knockout Mice

To determine how hearing loss progresses in Cep250 knockout mice, we measured the ABR thresholds in the Cep250 knockout mice. Cep250 knockout mice were obtained from Cyagen (C57BL/6N-*Cep250em1C*/Cya).

**Figure 5:**
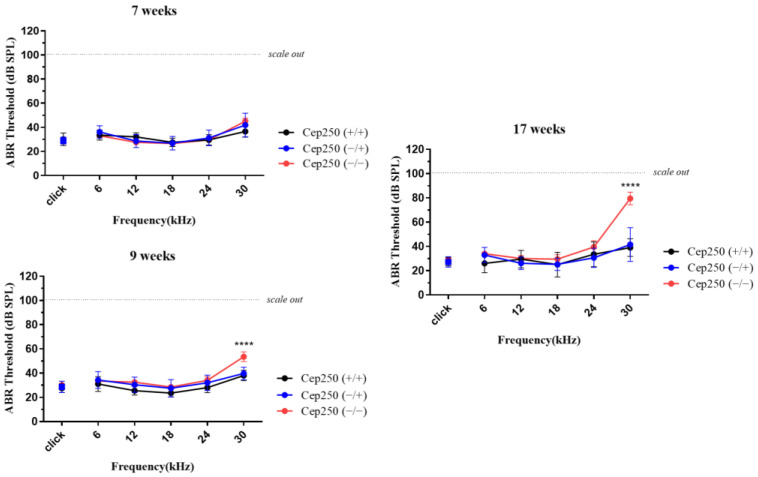
Auditory brainstem response (ABR) test in the *Cep250* knockout mice. ABR thresholds were measured at the stimuli of click and tone sounds. The number of *Cep250* (+/+), (+/−), and (−/−) mice was 5, 11, and 5, respectively. ****, *p* < 0.001.

The ABR test was performed at 7, 9, and 17 weeks of age. At the age of 7 weeks, there was no difference in ABR thresholds according to genotype. At the age of 9 weeks, *Cep250* (−/−) mice showed a significant increase in the auditory threshold at 30 kHz compared to *Cep250* (+/−) and *Cep250* (+/+) mice (Figure 5). Hearing loss at 30 kHz was more aggravated at 17 weeks of age in *Cep250* (−/−) mice.

### 3.6. Hair Cell Degeneration in the Cep250 Knockout Mice

**Figure 6:**
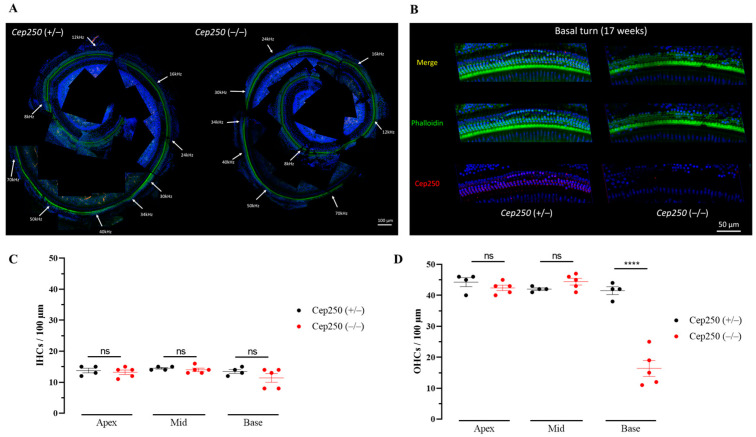
The cell survival rate in the *Cep250* knockout mice at the age of 17 weeks. (**A**) Whole-mount immunostaining with phalloidin (green), anti-Cep250 antibody (red), and Dapi (blue) was performed through the whole cochlear turn. Tonotopic frequencies are noted. (**B**) The basal turn of the *Cep250* knockout mouse showed a higher degeneration rate of the outer hair cell compared to that of WT mice. (**C**) Inner hair cell counts in apex, mid, and basal turns in CEP250 knockout mice. (**D**) Outer hair cell counts in apex, mid, and basal turns in *Cep250* knockout mice. IHC, inner hair cells; OHC, outer hair cells; ns, not significant; ****, *p* < 0.001.

To investigate the cochlear region responsible for the increased auditory threshold in the ABR test, we measured the hair cell survival rate in the *Cep250* knockout mice. Whole-mount immunostaining revealed that homozygous *Cep250* knockout mice showed more severe degeneration of hair cells than heterozygous *Cep250* mice (Figure 6A). In particular, outer hair cell degeneration was more robust in the high-frequency region of the basal turn (Figure 6B,C). Taken together, the progressive hearing loss at high frequencies in *Cep250* homozygous knockout mice is attributable to outer hair cell death, indicating that Cep250 in the cochlea is critical for maintaining outer hair cell survival in adult mice.

## 4. Discussion

In this study, we report, for the first time, that a homozygous nonsense variant of *CEP250* causes NSHL with autosomal recessive inheritance in humans. The novel variant *CEP250* c.3511C>T; p.Gln1171Ter was associated with slowly progressive sensorineural hearing loss, but was not related to ocular phenotypes such as cone-rod dystrophy or RP. In a subsequent molecular study, we found that the nonsense variant does not induce nonsense-mediated decay of mRNA, but is well translated into the protein; however, the CEP250 protein failed to localize in the centrosome. Using *Cep250* knockout mice, Cep250 was broadly expressed in hair cells and ganglion neurons. Without Cep250, the outer hair cells degenerate, and subsequently, auditory thresholds increase with age. Data from both humans and mice support that Cep250 plays an important role in maintaining hair cell function and survival in the cochlea during the postnatal period.

To date, there have been five reports on the association between *CEP250* pathogenic variants and atypical Usher syndrome or nonsyndromic RP [10,11,12,13,14]. Usher’s syndrome is associated with sensorineural hearing loss and RP [27]. Usher syndrome type 1 (USH1) causes congenital severe-to-profound hearing loss with vestibular dysfunction and early onset RP (first decade of life). Usher syndrome type II (USH2) is characterized by moderate congenital hearing loss without vestibular dysfunction and relatively late-onset RP (second decade of life). In Usher type III, progressive sensorineural hearing loss begins after speech development and varying degrees of RP and vestibular dysfunction are present [28,29,30,31]. Atypical Usher syndrome comprises those cases of Usher syndrome that cannot be categorized into the other three types (I, II, and III). Several studies have reported that ultra-rare variants of the causative genes are associated with atypical Usher syndrome. For instance, *MYO7A*, *USH2A*, *CDH23*, *USH1G*, *CEP250*, *CEP78*, *ADGRV1*, *ARSG*, *ABHD12*, and *ESPN* are associated with the atypical phenotypes of Usher syndrome [32]. The presence of an atypical phenotype indicates that Usher syndrome is a spectral disease with variable severity and multi-organ phenotypes. In this regard, the phenotype of patients with variants of *CEP250* may vary depending on the severity of damage to individual variants of *CEP250*. This speculation is in line with evidence that the severity of RP is dependent on the type of nonsense variants in *CEP250* and that a missense variant in *CEP250* is relevant to only the nonsyndromic RP [10,13,14]. In our study, patients with the *CEP250* p.Gln1171Ter variant did not have any ocular phenotype, but only had auditory phenotypes, such as progressive sensorineural hearing loss (i.e., NSHL). The ages of the affected individuals in this study ranged from the late 30 s to the early 40s, which is older than that reported previously for atypical Usher syndrome caused by variants in *CEP250* [32]. Nevertheless, we cannot exclude the possibility that retinal degeneration begins at a later age. According to the literature, *Cep250* knockout mice do not show visual phenotype at the ages of 3 and 6 months while their visual responses decrease at 12 and 20 months [33,34]. These findings support that visual phenotype may be age-dependent, and long-term follow-up is necessary. In the present study, we did not perform the test of visual response in the knockout mice. Two reports on *Cep250* knockout mice used the same knockout allele, which was generated by the deletion of exon 6 and 7, resulting in the early truncation of the 178 amino acid protein [33,34]. In our mice, we deleted exons 3–12 resulting in the early truncation of the 77 amino acid protein. As both strains generated a very early truncation of the protein and deleted exons 6 and 7, which are critical for Cep250 activity, we consider these two strains to be functionally equivalent in terms of *Cep250* loss-of-function; thus, we expect that our knockout mice would also show a similar pattern in visual phenotype. To determine whether the variant found in the patients shows a visual phenotype in a mouse model, it is needed to create a humanized knockin mouse model with the same variant as that found in the patients. This model would produce an early truncating protein of approximately 1171 amino acids, roughly half the size of the wild type CEP250 protein. As such, generating a knockin mice model and following its electroretinogram are warranted to delve deeper into investigating the pathologic mechanisms of the variant about retinal degeneration.

Most pathogenic variants of *CEP250* are nonsense recessive variants, indicating that early truncation of CEP250 results in loss of function. CEP250 mediates centriole cohesion, which is important for cilia development [35]. When we overexpressed WT CEP250 in NIH3T3 cells, ciliary development was normal (Figure 3). This finding indicated that the role of CEP250 in ciliary development may be redundant. This postulation is consistent with the late-onset and relatively mild phenotype of atypical Usher syndrome or nonsyndromic RP of hearing loss caused by variants in *CEP250*. Given that the onset of hearing loss occurs in adulthood, CEP250 in the cochlea may be involved in the maintenance of hair cell structure and function, but not in the developmental stage.

In our study, the nonsense variant did not undergo nonsense-mediated decay having vulnerable and easily degradable mRNAs. The nonsense variant mRNA (*CEP250* c.3511C>T) was successfully translated, but the protein failed to localize in the centrosome (Figure 2). However, early truncation of CEP250 did not affect ciliary elongation (Figure 3), indicating that the variant leads to loss of function but not gain of function. How the loss of function of CEP250 causes hair cell degeneration and leads to progressive hearing loss remains unclear. There are several possible explanations for this. First, given that CEP250 plays a role in centrosome formation and ciliary development by interacting with Rootletin and NEK2 [9,10], loss of function of CEP250 may cause defects in cellular proliferation and migration, which results in an unstable subcellular structure of hair cells, consequently causing progressive hearing loss. Second, an unknown function of CEP250 in hair cells may be required to maintain hair cell function and survival. CEP250 was abundantly expressed in hair cells and spiral ganglions (Figure 4). Without CEP250, it appears that the outer hair cells gradually degenerate, which could be either primary or secondary. These data suggest that CEP250 is critical for hair cell survival. Unfortunately, the present study does not provide the experimental data about whether primary cilia and centrosome are affected or not in the hair cells of *Cep250* knockout mice. Therefore, mechanistic investigation about defects in primary cilia and mislocalization of the centrosome would be helpful to elucidate the differential functions of CEP250 in hair cells in future studies.

## 5. Conclusions

A novel nonsense variant of *CEP250* (c.3511C>T) was identified in patients with nonsyndromic progressive hearing loss with an autosomal recessive inheritance pattern. The early truncated protein CEP250 (p.Gln1171Ter) showed a deficit in localization toward the centrosome. Given that Cep250 is expressed in the cochlear hair cells and spiral ganglion, defects in the centrosomal localization of CEP250 by the nonsense variant of *CEP250* may result in hearing deterioration.

## Data Availability

Data are contained within the article.

## Supplementary Materials

The following supporting information can be downloaded at: https://www.mdpi.com/article/10.3390/cells12182328/s1, Supplementary Table S1. Variants detected in YUHL251individual with hearing loss using whole exome sequencing.
