# Supplementary material for: Novel Variant in CEP250 Causes Protein Mislocalization and Leads to Nonsyndromic Autosomal Recessive Type of Progressive Hearing Loss
Source: Cells. 2023 Sep 21;12(18):2328. doi: 10.3390/cells12182328 (PMC10528078; doi:10.3390/cells12182328)
Supplement: Supplementary file 1 [file cells-12-02328-s001.zip › cells-2551147-supplementary.pdf]

Supplementary Table S1. Variants detected in YUHL251individual with hearing loss using whole exome sequencing.

Gene	Accession	Nucleotide change	Amino acid change	Exon	Zygo -sity	Amino acid sequence conserva -tion	dbSNP 154[a]	TOPMed[b]	gnomAD[c]	gnomAD EAS[d]	PP2[e]	MT[f]	SIFT[g]	CADD[h]	ClinVar[i]
CEP250	NM_007186.6	c.3511C>T	p.Gln1171*	26/35	Hom	NA	ND	ND	ND	ND	ND	A(1)	ND	36	ND
PECR	NM_018441.6	c.491G>T	p.Gly164Val	4/8	Het	*Danio rerio*	ND	ND	ND	ND	Dam (1)	DC (1)	Del (0)	31	ND
		c.293T>G	p.Phe98Cys	3/8	Het	*Macaca mulatta*	rs20131 9769	0.000023	3.58E-05	0.0004349	Dam (0.951)	Neu (0.74)	Del (0)	24.1	ND
EXD3	NM_017820.5	c.727C>T	p.Arg243Cys	8/22	Het	*Macaca mulatta*	rs20053 8457	ND	0.000506 7	5.80E-05	Dam (0.791)	DC (0.86)	Del (0.01)	23.1	ND
		c.482C>T	p.Thr161Met	6/22	Het	*Macaca mulatta*	rs55538 3988	ND	1.63E-05	5.57E-05	Ben (0.266)	DC (0.99)	Del (0.01)	15.99	ND
GATA1	NM_002049.4	c.212A>C	p.His71Pro	2/6	Hom	*Mus musculus*	rs37430 0356	ND	6.87E-05	0.000864	Ben (0.178)	DC (0.99)	Del (0)	20.8	Benign
CTNNBL1	NM_030877.5	c.1646G>A	p.Arg549Gln	16/16	Hom	*Mus musculus*	rs20188 7283	0.000060	0.000123 4	0.001033	Ben (0.005)	DC (0.99)	Tol (0.16)	22.6	ND
LRRC32	NM_005512.3	c.1393C>T	p.His465Tyr	3/3	Het	*Mus musculus*	rs14274 88745	ND	ND	ND	Ben (0.206)	Neu (0.99)	Tol (0.08)	20.4	ND
		c.839C>T	p.Pro280Leu	3/3	Het	*Mus musculus*	rs11633 30112	0.000015	7.96E-06	0.0001087	Ben (0.003)	Neu (0.99)	Tol (0.2)	16.05	ND

Dam, probably damaging; Del, deleterious; DC, disease-causing; A, disease-causing automatic; Ben, benign; Neu, neutral; Tol, tolerated; Het, heterozygous in the affected individual; Hom, homozygous in the affected individual; ND, no data; NA, not applicable.

[a]dbSNP database (http://www.ncbi.nlm.nih.gov/SNP).
[b]NHLBI Trans-Omics for Precision Medicine (TOPMed) (https://topmed.nhlbi.nih.gov/)
[c]Population frequency of variants in gnomAD (https://gnomad.broadinstitute.org/).
[d]East Asian Population frequency of variants in gnomAD (https://gnomad.broadinstitute.org/).
[e]PolyPhen-2 HumVar prediction score (http://genetics.bwh.harvard.edu/pph2/).
[f]MutationTaster (http://www.mutationtaster.org/).
[g]SIFT Sorting Intolerant from Tolerant (http://sift.jcvi.org/).
[h]phred-like scores (scaled C-scores) on the Combined Annotation-Dependent Depletion (http://cadd.gs.washington.edu/home/).
[i]Clinvar, public archive with free access to reports on the relationships between human variations and phenotypes, with supporting evidence. (https://www.ncbi.nlm.nih.gov/clinvar/).
